# High-Density Lipoprotein Cholesterol: A Component of the Metabolic Syndrome with a New Role in Lung Function

**DOI:** 10.1155/2021/6615595

**Published:** 2021-06-02

**Authors:** Faxuan Wang, Di Tian, Yi Zhao, Jiangping Li, Xiyuan Chen, Yuhong Zhang

**Affiliations:** ^1^School of Public Health and Management, Ningxia Medical University, Yinchuan, China; ^2^Key Laboratory of Environmental Factors and Chronic Disease Control, No. 1160, Shengli Street, Xingqing District, Ningxia, Yinchuan, China

## Abstract

**Background:**

A considerable uncertainty exists about the relationship between adult metabolic syndrome (MS) and obstructive lung disease (OLD), perhaps owing to systemic inflammation. Therefore, this study aimed to investigate the relationship between MS (with its components) and the patterns of lung function impairment.

**Methods:**

The participants in this study were 3978 adults aged 30-78 years from the baseline cohort of the Ningxia Cohort Study. The participants underwent pulmonary function tests, questionnaire surveys, physical examinations, and analysis of blood specimens.

**Results:**

No significant difference in the prevalence of OLD was observed between male (15.9%) and female (14.2%) participants. After adjusting for possible confounding factors (e.g., age and family income), impaired lung function was found to be related to some MS components, such as abdominal obesity, high blood pressure, and low levels of high-density lipoprotein cholesterol (HDL-C) (all *P* < 0.05).

**Conclusions:**

As an important component of MS, abdominal obesity is related to impaired lung function. Surprisingly, this study found that increased HDL-C levels could accelerate the decline of lung function; it also suggests that in the presence of different metabolic health conditions, especially abdominal obesity and low levels of HDL-C, various metabolic indicators should be comprehensively considered to prevent the decline of lung function. This partly explains the increase in the incidence of two or more chronic diseases. Therefore, the prevention of chronic diseases should shift from single-disease prevention to a comprehensive consideration of multi-disease prevention in the future. Therefore, a more sensitive evaluation of the role of HDL-C in lung function is warranted.

## 1. Introduction

Chronic obstructive pulmonary disease (COPD) generally develops progressively. It is characterized by an incomplete and reversible restriction of airflow and is related to abnormal inflammatory reactions in the lungs caused by factors such as harmful particles or gases. COPD is one of the causes of increased morbidity and mortality worldwide [1]. In addition, COPD is expected to become the sixth leading cause of death globally by 2017 [2]. In China, COPD has a prevalence of 8.2% in people older than 40 years and is the fifth leading cause of death [3, 4]. Therefore, the identification and assessment of COPD and other related diseases are important to prevent disease progression.

In the diagnosis of COPD, a term that encapsulates its inflammatory nature and comorbid conditions is “chronic systemic inflammatory syndrome” [5, 6]. The metabolic syndrome (MS) may be one of its comorbid conditions. MS is characterized by insulin resistance, abdominal obesity, hypertension, and dyslipidemia, which can increase the risk of cardiovascular disease and type 2 diabetes [7, 8]. One of the symptoms of COPD is lung dysfunction [9]. Recent studies on the role of MS and its components in predicting lung dysfunction have received special attention [10–12].

Studies in Italy, Taiwan, and Japan have shown that MS is independently associated with restrictive patterns of impaired lung function [13–15], and other reported results have also indicated that MS is related to restrictive lung disease (RLD). In contrast, a cohort study in Guangzhou, China, has found that MS is associated with airflow obstruction [16]. Meanwhile, another study suggested that airflow obstruction of Global Initiative for Chronic Obstructive Lung Disease stage II-IV might be associated with MS in Japanese men [17]. However, only a few large-scale studies have investigated the relationship between obstructive lung disease (OLD) and MS. Therefore, this study aimed to investigate the relationship between MS (with its components) and lung function in adults of both sexes in rural China.

## 2. Materials and Methods

### 2.1. Study Setting

**Figure 1:**
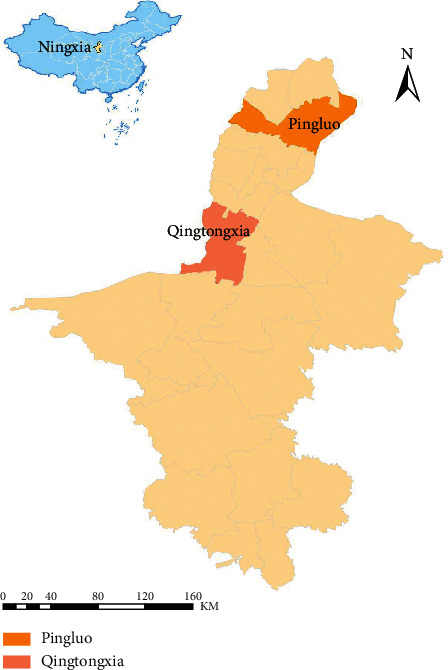
Locations of Pingluo and Qingtongxia counties in Ningxia Hui Autonomous Region, China.

Figure 1 shows the locations of Pingluo (in the north) and Qingtongxia (in the middle) in the Ningxia Hui Autonomous Region. The two counties have an area of approximately 2060 and 2525 km^2^, respectively. The climate is characterized by a long duration of cold winter weather, a short duration of hot summer weather, and a dry weather with less rain and snow.

### 2.2. Study Design and Participants

**Figure 2:**
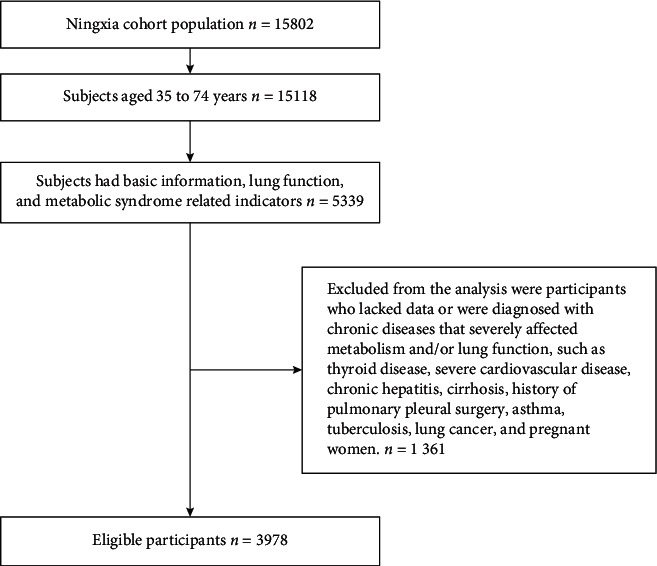
Selection of participants for inclusion in the study.

This study used the baseline data of the Ningxia Cohort Study from the China Northwest Cohort, which was performed in Pingluo and Qingtongxia in Ningxia from March 2018 to May 2019. All participants met the following inclusion criteria: (i) age 30-78 years, (ii) a registered permanent residence in the survey location that is inhabited for > 5 months in a year, (iii) normal communication ability without serious problems such as physical disability, and (iv) personal social medical insurance coverage from the local health department. The specific process of participant inclusion in the study is shown in Figure 2.

This study was based on township. The health personnel of the study township were responsible for daily routine services and promoted communication with all participants in the cohort. After an overnight fast, the participants were invited to the township health hospital for a questionnaire survey and physical examination.

The research protocol was approved by the Ethical Committee of Ningxia Medical University (no. 2018-012). All eligible participants understood the specific process of the study. All participants provided written informed consent for follow-up, participation, storage and use of blood and urine samples, and use of medical record information.

### 2.3. Data Collection and Measurement

The questionnaire survey was conducted by trained university students and rural doctors. During the health evaluation, the basic information of the participants, including age, educational level, family income, smoking status, alcohol/drinking status, and family disease history (e.g., heart disease, high blood pressure, or asthma), was collected through the face-to-face method. The participants were classified as “heavy smokers” (those who reported smoking more than one cigarette a day for more than 6 months) or “light smokers” (those who reported not smoking more than one cigarette per day and for no more than 6 months). With respect to alcohol drinking, the participants were divided into “heavy drinkers” (those who reported drinking alcohol at least once a week for more than 6 months) or “light drinkers” (those who reported drinking alcohol less than once a week on average). Educational level was divided into primary and nonformal school education, junior high school, and senior high school or above. The family income groups were set according to quartiles: “quartile 1” represented the lowest family income status and “quartile 4” represented the highest family income status.

Fasting plasma glucose (FPG), triglyceride (TG), and high-density lipoprotein cholesterol (HDL-C) data were obtained from the laboratory of the local health hospital, which tested the participants' blood samples. Height, weight, and waist circumference (WC) were evaluated by trained personnel using a bioelectrical impedance analyzer (InBody 370; InBody, Seoul, South Korea) with eight-point tactile electrodes. The analyzer was calibrated every morning before the inspection, and the repeatability and accuracy of body composition data were checked. Blood pressure was measured using an upper-arm electronic sphygmomanometer, three times after the participants had rested in a sitting position for 5 min. Body mass index (BMI) was calculated by dividing weight (kg) by the square of height (m^2^).

### 2.4. Assessment of Lung Function

Lung function parameters were measured using a digital spirometer connected to a computer (Chestgraph HI-101; Chest Ltd., Tokyo, Japan), including forced expiratory volume in 1 s (FEV1) and forced vital capacity (FVC). The spirometer was able to determine the predicted FEV1 and FVC values according to the following prediction equations:

(1) $$\begin{array}{c} \text{FVC}\left(\text{mL}\right)=\left(2.94-0.010\times \text{age}\left[\text{years}\right]\right)\times \left(\;\frac{\text{height}\left[\text{cm}\right]}{100}\right)\left(\text{men}\right),\\ \text{FVC}\left(\text{mL}\right)=\left(2.33-0.008\times \text{age}\left[\text{years}\right]\right)\times \left(\;\frac{\text{height}\left[\text{cm}\right]}{100}\right)\left(\text{women}\right),\\ \text{FEV}1\left(\text{mL}\right)=\left(2.72-0.015\times \text{age}\left[\text{years}\right]\right)\times \left(\;\frac{\text{height}\left[\text{cm}\right]}{100}\right)\left(\text{men}\right),\\ \text{FEV}1\left(\text{mL}\right)=\left(2.19-0.013\times \text{age}\left[\text{years}\right]\right)\times \left(\;\frac{\text{height}\left[\text{cm}\right]}{100}\right)\left(\text{women}\right). \end{array}$$

The predicted FVC and FEV1 values vary with the characteristics of a given population (age, height, sex, and race/ethnicity). According to the recommendations of the American Thoracic Society [18], the spirometer needs to be calibrated according to the manufacturer's instructions before each measurement. The participants were asked to wear a nose clip while maintaining a sitting posture on a chair. All lung function tests were completed through the mouthpieces. All participants were required to produce three curves that meet the standard, and the best value was used for analysis. The spirometer was used every morning after calibration, according to the manufacturer's instructions. We defined RLD as FVC <80% of the predicted value and FEV1/FVC ratio >70%. We defined OLD as FEV1/FVC ratio <70%.

### 2.5. Assessment of MS

We classified the participants as having MS if they had abdominal obesity, as defined by the International Diabetes Federation diagnostic criteria [19] (WC ≥ 90 cm for men and ≥80 cm for women), plus any two of the following four factors according to the National Cholesterol Education Program Adult Treatment Panel III [20] definition:

- FPG ≥ 5.6 mmol/L or previously diagnosed type 2 diabetes.
- TG ≥ 1.7 mmol/L or specific treatment for this lipid abnormality.
- HDL-C < 1.03 mmol/L in men or < 1.29 mmol/L in women or specific treatment for this lipid abnormality.
- Systolic blood pressure (SBP) ≥ 130 mmHg or diastolic blood pressure (DBP) ≥ 85 mmHg or treatment of previously diagnosed hypertension.

Participants without any of the five risk factors mentioned above received an MS score of 0, and those with one, two, three, and four or more of the risk factors received an MS score of 1, 2, 3, and ≥4, respectively.

### 2.6. Statistical Analysis

The results are expressed as mean ± standard deviation, and categorical variables are expressed as frequency. The chi-square test was used to assess the differences in categorical variables between the sexes, and one-way analysis of variance was used to assess continuous variables. Multiple linear regression models were established to evaluate the relationship between lung function and metabolic components. Logistic regression models were established to evaluate the relationship between two lung diseases and MS and its scores, and two models were adjusted for potential confounding factors including age, smoking, drinking, educational level, family income group, and BMI. All analyses were performed using SPSS (version 23.0; SPSS Inc., Chicago, IL, USA). A two-sided *P* value of <0.05 was considered to indicate statistical significance.

## 3. Results and Discussion

### 3.1. Results

A total of 3978 participants (1760 men and 2218 women) were included in this study (Table 1). The average age of female participants was lower than that of male participants (mean age 53.6 ± 8.3 vs. 58.1 ± 8.8 years, *P* < 0.001). In this study, men were generally more educated and had a relatively higher frequency of smoking and drinking than women, with statistical differences. However, in terms of family income, there was no statistical difference between men and women. Except for TG (*P*=0.493), there were significant differences between the sexes in the other evaluated clinical characteristics (*P* < 0.001). Women had lower WC (86.2 ± 8.8 vs. 88.9 ± 10.2 cm), SBP (133.3 ± 18.5 vs. 135.1 ± 18.3 mmHg), DBP (82.2 ± 11.7 vs. 83.8 ± 12.4 mmHg), and FPG (5.4 ± 0.8 vs. 5.5 ± 0.8 mmol/L), but higher HDL-C (1.4 ± 0.3 vs. 1.3 ± 0.3 mmol/L), than men. The prevalence of MS in men was significantly lower than that in women. Although there was no statistical difference between the average estimated value of FEV1 (% predicted) and the ratio of FEV1/FVC between the sexes, the average estimated value of FEV1 was significantly lower in women. There were more male participants with OLD than female participants. In contrast, the number of men with RLD was lower than that of women, with no significant difference between the sexes.

Data are shown as mean ± standard deviation or *n* (%). BMI, body mass index; WC, waist circumference; SBP, systolic blood pressure; DBP, diastolic blood pressure; FPG, fasting plasma glucose; TG, triglycerides; HDL-C, high-density lipoprotein cholesterol; MS, metabolic syndrome; FVC, forced vital capacity; FEV1, forced expiratory volume in 1 s; OLD, obstructive lung disease; RLD, restrictive lung disease.

The relationship between the various components of MS and the lung function status is shown in Table 2. After multiple linear regression analysis, among men, DBP and FPG had a significant positive correlation with FVC, whereas WC and SBP had an opposite or a negative correlation. Among women, a weaker association between DBP and FVC was observed. Among all participants, a significant negative correlation was found between HDL-C and FVC. WC, SBP, DBP, and FEV1 showed a similar relationship with FVC in men. There was a strong negative correlation between HDL-C level and FEV1 in women, whereas the other variables were not correlated. However, HDL-C was weakly correlated with the FEV1/FVC ratio in men. None of the variables in women were related to FEV1.

In Table 3, the prevalence of MS with RLD (38.2% vs. 32.9%) and OLD (48.7% vs. 40.8%) was significantly different between the sexes. After adjusting the crude model and models 1 and 2, among all participants, there was no significant difference between RLD and MS. In addition, after adjusting the crude model and models 1 and 2, there was no significant difference between OLD and MS in men. However, there were significant differences between OLD and MS in the crude model (0.695, 95% confidence interval [CI] 0.546-0.886) or in model 1 (0.682, 95% CI 0.534-0.872) and model 2 (0.738, 95% CI 0.568-0.959) in women.

Data were calculated using multiple logistic regression. RLD, restrictive lung disease; OLD, obstructive lung disease; OR, odds ratio; CI, confidence interval. Model 1 was adjusted for age, smoking, and alcohol drinking. Model 2 was adjusted for age, smoking, alcohol drinking, educational level, family income group, and body mass index.

The results of the analysis of the relationship between OLD and MS scores are shown in Table 4. Among the participants, the number of men with MS scores of 1, 2, 3, and 4+ was 74, 87, 61, and 31, respectively. The corresponding numbers for women were 65, 98, 80, and 48, respectively. In women, OLD and an MS score of 4 were statistically significant in the crude model (0.539, 95% CI 0.314-0.925) and in the adjusted model 1 (0.507, 95% CI 0.294-0.873). However, after adjusting for confounding factors, the relationship between OLD and the MS score was not statistically significant among men.

Data were calculated using multiple logistic regression. OLD, obstructive lung disease; MS, metabolic syndrome; OR, odds ratio; CI, confidence interval. Model 1 was adjusted for smoking and family income group. Model 2 was adjusted for age, smoking, alcohol drinking, educational level, family income group, and body mass index.

## 4. Discussion

In this study, abdominal obesity, low levels of HDL, and high blood pressure, which are components of MS, were significantly associated with decreased FVC and FEV1. Furthermore, MS and an MS score of 4 had a negative correlation with OLD in women.

When each metabolic component was used to independently predict FVC (% predicted value), FEV1 (% predicted value), and the FEV1/FVC ratio, the result was consistent with that of previous studies [14, 21, 22]: after adjusting for age and family income group, WC was one of the MS components related to FVC and FEV1 reduction among men. To date, central obesity (abdominal obesity) is considered to be a common cause of metabolic and cardiovascular diseases in adults, including hyperglycemia, hypertension, and dyslipidemia. Central obesity is also considered the main component of MS [23]. Recent studies have mostly shown that the connection between MS and a weakened lung function is mainly based on abdominal obesity. WC is one of the diagnostic indicators of abdominal obesity, which is related to the decline of lung function [24]. Excessive abdominal fat in patients with central obesity might limit the expansion of the diaphragm, producing a mechanical effect on the lungs. As there are sex differences in the respiratory movement and fat distribution of the chest wall and abdominal wall, the impact on lung function also differs between men and women [25]. Furthermore, MS is characterized by systemic inflammation, which results from endothelial dysfunction that can lead to impaired organ system function [26, 27]. Moreover, this study found that there were significantly more male smokers than female smokers, and it has been reported that systemic inflammation can be caused by long-term exposure to cigarette smoke [28]. Therefore, among active smokers with MS, there are two sources of inflammation: visceral fat and lung exposure to cigarette smoke. The combined effect of these two sources may increase the occurrence of systemic inflammation, which can lead to an increase in endothelial dysfunction and a rapid decline in lung function.

Notably, HDL-C is an important component of MS and its increase was significantly related to the decrease in FVC and FEV1 in the participants of this study. A study in a representative sample in the United States reported results consistent to those generated in this study and suggested that low levels of HDL-C is associated with impaired lung function [12]. A small case-control study on the relationship between MS and COPD showed that the HDL level of the COPD group was crucially lower than that of the control group (47.1% vs 58%) [29]. Moreover, a small population study [30] found that participants with normal or high levels of HDL-C had lower FVC than those with low levels of HDL-C. The pathophysiological role between the two is still uncertain. It is generally believed that chronic inflammation can accelerate atherosclerosis, which is achieved by changing the HDL level and its capability to advance reverse cholesterol transport [31, 32]. Because this lipoprotein has anti-fungal, anti-inflammatory, anti-oxidant, and even anti-apoptotic functions, it is expected to play a beneficial role in lung function [33–35]. However, another study suggested that although there was a negative association between HDL-C and FEV1 and FVC, it cannot indicate lung involvement, despite the statistical significance [36].

However, the role of HDL-C has become more clearly understood in recent years, especially as a protective factor against atherosclerosis. In fact, it has been realized that the ability to resist inflammation and to mobilize cholesterol is significantly affected by the oxidation of HDL-related proteins and that even dysfunctional HDL may also have pro-inflammatory effects. This new understanding of the physiological function of HDL-C may partially explain the result of this study (i.e., the higher the HDL-C level, the smaller the lung capacity) [37]. Meanwhile, a genetic and molecular study showed that changes in the gene expression of apolipoprotein *M* (APOM), a lipoprotein related to HDL-C, can change the quality and function of HDL-C. The study found that two polymorphisms of APOM-related genes are related to lung function decline in two ethnic groups (African-American and European-American). Further, studies have also found that high levels of HDL-C are related to a decrease in the FEV1/FVC ratio [38].

Contrary to previous studies, this study found that MS and OLD were negatively correlated in women, whereas the MS score was negatively correlated with the incidence of OLD [16, 39, 40]. However, after a careful observation of the relationship between MS components and OLD, this study found that HDL-C plays a more vital role than other metabolic components and is notably negatively correlated with FEV1 and FVC in women. This result is consistent with a previous study that found that, in a model without variable adjustment, Japanese patients with stage I airflow obstruction (FEV1/FVC < 70%, FEV1 ≥ 80% of predicted value) were at a lower risk of MS than patients with normal lung function [17].

The findings of this study highlight the connection between MS and its components (especially abdominal obesity and low levels of HDL-C) and lung function in the Chinese population. Therefore, in the case of impaired lung function, the possibility of this relationship should not be overlooked. However, this study had some limitations. This was a cross-sectional study of the relationship between MS and its components and decreased lung function. Owing to this study design, a causal relationship cannot be determined. In addition, although many possible variables were selected in this study, it is inevitable that some other variables were omitted.

## 5. Conclusions

The current study shows that MS and its components are related to impaired lung function, and the intensity and direction of this relationship vary. This finding suggests that in the presence of different metabolic health conditions, especially abdominal obesity and low levels of HDL-C, various metabolic indicators should be comprehensively considered to prevent the decline of lung function. This partly explains the increase in the incidence of two or more chronic diseases. Accordingly, the prevention of chronic diseases should shift from single-disease prevention to a comprehensive consideration of multi-disease prevention in the future.

## Figures and Tables

**Table 1:** Basic characteristic of the study participants.

	Men (*n* = 1760)	Women (*n* = 2218)	All (*n* = 3978)	*P* value
Age (years)	58.1 ± 8.8	53.6 ± 8.3	55.6 ± 8.8	<0.001
Educational level (%)				<0.001
Primary and nonformal school education	1026 (58.3)	1463 (66.0)	2489 (62.6)	
Junior high school	625 (35.5)	672 (30.3)	1297 (32.6)	
Senior high school or above	109 (6.2)	83 (3.7)	192 (4.8)	
Family income group (%)				0.087
Quartile 1 (<¥10,000)	400 (22.7)	449 (20.2)	849 (21.3)	
Quartile 2 (¥10,000-¥20,000)	507 (28.8)	610 (27.5)	1117 (28.1)	
Quartile 3 (¥20,000-50,000)	398 (22.6)	525 (23.7)	923 (23.2)	
Quartile 4 (>¥50,000)	455 (25.9)	634 (28.6)	1089 (27.4)	
Smoking				<0.001
Heavy (%)	498 (28.3)	11 (0.5)	509 (12.8)	
Light (%)	1262 (71.7)	2207 (99.5)	3469 (87.2)	
Alcohol drinking				<0.001
Heavy (%)	261 (14.8)	21 (0.9)	282 (7.1)	
Light (%)	1499 (85.2)	2197 (99.1)	3696 (92.9)	
BMI (kg/m^2^)	25.0 ± 3.3	24.8 ± 3.2	24.9 ± 3.3	0.148
WC (cm)	88.9 ± 10.2	86.2 ± 8.8	87.4 ± 9.5	<0.001
SBP (mmHg)	135.1 ± 18.3	133.3 ± 18.5	134.1 ± 18.4	0.002
DBP (mmHg)	83.8 ± 12.4	82.2 ± 11.7	82.9 ± 12.1	<0.001
FPG (mmol/L)	5.5 ± 0.8	5.4 ± 0.8	5.5 ± 0.8	<0.001
TG (mmol/L)	1.5 ± 0.8	1.5 ± 0.7	1.5 ± 0.7	0.493
HDL-C (mmol/L)	1.3 ± 0.3	1.4 ± 0.3	1.3 ± 0.3	<0.001
MS (%)	664 (37.7)	1075 (48.5)	1739 (43.7)	<0.001
Abdominal obesity	768 (43.6)	1680 (75.7)	2448 (61.5)	<0.001
High blood pressure	1187 (67.4)	1324 (59.7)	2511 (63.1)	<0.001
High glucose	727 (41.3)	780 (35.2)	1507 (37.9)	<0.001
High triglycerides	624 (35.4)	802 (36.2)	1426 (35.8)	0.646
Low levels of HDL-C	372 (21.1)	849 (38.3)	1221 (30.7)	<0.001
FVC % predicted	75.5 ± 15.5	77.5 ± 15.4	76.6 ± 15.5	<0.001
FVC (L)	2.7 ± 0.6	2.1 ± 0.5	2.4 ± 0.6	<0.001
FEV1 % predicted	75.2 ± 15.2	74.2 ± 14.9	74.6 ± 15.0	0.043
FEV1 (L)	2.1 ± 0.5	1.7 ± 0.4	1.9 ± 0.5	<0.001
FEV1/FVC ratio	80.1 ± 11.8	80.9 ± 11.5	80.6 ± 11.7	0.198
OLD (%)	280 (15.9)	314 (14.2)	594 (14.9)	0.068
RLD (%)	883 (50.2)	1071 (48.3)	1954 (49.1)	0.125

**Table 2:** Regression coefficients of each metabolic component individually entered into adjusted models for predicting FVC (% predicted), FEV1 (% predicted), and FEV1/FVC ratio.

	FVC (% predicted)		FEV1 (% predicted)		FEV1/FVC ratio	
	Men	Women	Men	Women	Men	Women
Variables	*β* (SE)	*β* (SE)	*β* (SE)	*β* (SE)	*β* (SE)	*β* (SE)
WC (cm)	−0.067 (0.038)^*∗∗*^	0.041 (0.039)	−0.057 (0.039)^*∗*^	0.037 (0.038)	0.041 (0.030)	0.007 (0.030)
SBP (mmHg)	−0.070 (0.028)^*∗*^	−0.058 (0.028)	−0.080 (0.029)^*∗*^	−0.017 (0.028)	−0.005 (0.023)	0.030 (0.022)
DBP (mmHg)	0.100 (0.040)^*∗∗*^	0.068 (0.042)^*∗*^	0.068 (0.041)^*∗*^	0.037 (0.042)	−0.028 (0.032)	−0.014 (0.032)
FPG (mmol/L)	0.056 (0.450)^*∗*^	0.034 (0.429)	0.028 (0.459)	0.007 (0.424)	−0.032 (0.359)	−0.014 (0.329)
TG (mmol/L)	−0.003 (0.499)	−0.028 (0.464)	0.032 (0.509)	−0.007 (0.459)	0.038 (0.398)	0.021 (0.357)
HDL-C (mmol/L)	−0.068 (1.233)^*∗∗*^	−0.136 (1.120)^*∗∗∗*^	−0.029 (1.257)	−0.100 (1.109)^*∗∗∗*^	0.054 (0.984)^*∗*^	0.013 (0.861)

Data were calculated using multiple linear regression. Adjustments were made for age and family income group. WC, waist circumference; SBP, systolic blood pressure; DBP, diastolic blood pressure; FPG, fasting plasma glucose; HDL-C, high-density lipoprotein cholesterol; TG, triglycerides; FVC, forced vital capacity; FEV1, forced expiratory volume in 1 s; SE, standard error. ^*∗*^*P* < 0.05, ^*∗∗*^*P* < 0.01, and ^*∗∗∗*^*P* < 0.001.

**Table 3:** Odds ratios for RLD and OLD in metabolic syndrome.

Variable	RLD		OLD	
	Men	Women	Men	Women
MS (%)	38.2	48.7	32.9	40.8
Crude OR (95% CI)	1.038 (0.856-1.259)	1.021 (0.865-1.207)	0.777 (0.593-1.018)	0.695 (0.546-0.886)
Adjust OR (95% CI)				
Model 1	1.043 (0.858-1.268)	0.942 (0.794-1.116)	0.772 (0.589-1.013)	0.682 (0.534-0.872)
Model 2	0.968 (0.777-1.206)	0.927 (0.773-1.111)	0.825 (0.609-1.117)	0.738 (0.568-0.959)

**Table 4:** Association between OLD and MS score in men and women according to odd ratios.

	Men				Women			
	Crude model	Model 1	Model 2	Crude model	Model 1	Model 2
MS score	n (%)	OR (95% CI)	OR (95% CI)	OR (95% CI)	n (%)	OR (95% CI)	OR (95% CI)	OR (95% CI)
0	27 (9.6)	Reference	Reference	Reference	23 (7.3)	Reference	Reference	Reference
1	74 (26.4)	1.253 (0.776-2.025)	1.259 (0.778-2.039)	1.284 (0.791-2.084)	65 (20.7)	0.972 (0.576-1.639)	0.936 (0.554-1.582)	0.984 (0.578-1.675)
2	87 (31.7)	1.206 (0.754-1.929)	1.211 (0.754-1.943)	1.285 (0.786-2.101)	98 (31.2)	0.875 (0.531-1.440)	0.837 (0.507-1.381)	0.920 (0.544-1.556)
3	61 (21.8)	0.936 (0.573-1.529)	0.935 (0.572-1.530)	1.035 (0.608-1.762)	80 (25.5)	0.722 (0.435-1.200)	0.699 (0.420-1.163)	0.792 (0.457-1.372)
≥4	31 (11.1)	0.899 (0.515-1.569)	1.091 (0.820-1.452)	1.029 (0.557-1.901)	48 (15.3)	0.539 (0.314-0.925)	0.507 (0.294-0.873)	0.577 (0.321-1.037)

## Data Availability

The data used to support the findings of this study are available from the corresponding author upon request. Yuhong Zhang: zhabour@163.com.

## Abbreviations

COPD: Chronic obstructive pulmonary disease
MS: Metabolic syndrome
OLD: Obstructive lung disease
RLD: Restrictive lung disease
BMI: Body mass index
WC: Waist circumference
SBP: Systolic blood pressure
DBP: Diastolic blood pressure
FPG: Fasting plasma glucose
TG: Triglycerides
HDL-C: High-density lipoprotein cholesterol
FVC: Forced vital capacity
FEV1: Forced expiratory volume in 1 s.
